# Editorial: Rising stars: Clinical diabetes 2021

**DOI:** 10.3389/fendo.2022.1106804

**Published:** 2022-12-07

**Authors:** Ashraf Al Madhoun, Hidetaka Hamasaki

**Affiliations:** ^1^ Genetics and Bioinformatics, Dasman Diabetes Institute, Dasman, Kuwait; ^2^ Animal and Imaging Core Facilities, Dasman Diabetes Institute, Dasman, Kuwait; ^3^ Hamasaki Clinic, Kagoshima, Japan

**Keywords:** diabetes mellitus, T2D, T1D, inflammatory factors, meta-analysis

The global prevalence of diabetes mellitus (DM) has increased dramatically over the last few decades; due to which, the healthcare community has been exploring measures to prevent DM. DM is a metabolic disorder and there are miscellany factors contributing toward its cause; among which, the intertwined roles of genetic and environmental factors remain to be further elucidated. Therefore, personalized treatment interventions and changes in healthcare policies addressing the emerging needs of patients with DM need to be more effective. The complex pathophysiology of DM involves the disruption of critical factors, including glucose homeostasis, insulin resistance, and insulin production and secretion. There are different types of DM, including type 1 diabetes (T1D), type 2 diabetes (T2D), and gestational diabetes mellitus (GDM). Other classes have also been described, and the field is open for further investigations; however, this is beyond the topic of this editorial.

Identifying biochemical markers associated with the risk of T2D for early detection is crucial to prevent disease progression. With this aim, Bi et al. clarified the association between gamma-glutamyl transferase (GGT) and T2D using real-world data, Mendelian randomization (MR) analysis, and literature mining. GGT is a cell membrane enzyme that catalyzes glutathione and is associated with several metabolic disorders. Although it is produced in several tissues, its release into the serum is mainly derived from the liver. In a cohort of 3048 participants, Bi et al. discovered that GGT was correlated with indicators of glucose metabolism, including fasting plasma glucose and glycosylated hemoglobin (HbA1c) levels. In contrast to the notion that GGT is indicative of T2D development, MR analysis failed to provide exclusive evidence stating that the enzyme is a causal factor of T2D. Therefore, the authors suggested further investigation to determine whether GGT has a non-linear causal effect on T2D.

Giving the implications of pro-inflammatory factors in the progression of T2D, Aldadul et al. conducted a systematic review and meta-analysis, following the Preferred Reporting Items for Systematic Review and Meta Analyses (PRISMA) guidelines, to encapsulate the role of interleukin-1β in pancreatic β-cell deterioration. The authors found 13 research articles, published within the past five years, that described the association between T2D and circulating IL-1β. These studies included a total of 1182 patients with T2D (mean age: 54.2 ± 6.7 years) and 1498 controls (mean age: 50.2 ± 6.2 years). Protein and mRNA levels of plasma IL-1β were assessed in 12 and 4 studies, respectively. Although, the protein and mRNA levels of IL-1β in plasma were notably elevated in patients with T2D and were directly proportional to fasting plasma glucose and Hb1Ac levels, there was inconsistency between the findings of the reported studies. The authors attributed this to the limited number of studies and concluded that plasma levels of IL-1β are high, but nonsignificant, in patients with T2D. Furthermore, the meta-analysis highlighted difficulties caused by the absence of a standard protocol to assay plasma IL-1β levels, along with differences in sample size, population characteristics, and experimental design. Finally, the authors recommended large scale observational and longitudinal studies to precisely elucidate the role of IL-1β as a biomarker for T2D.

T2D adversely affects health-related quality of life of patients. One of the many complications of T2D is diabetic foot ulcer (DFU), which not only reduces the patients’ ability to carry out physical and social activities, but an absence of immediate intervention may cause diabetic foot attack (DFA). DFA is defined as an acute progressive necrosis of the foot’s soft and bone tissue, resulting in infection, hyperthermia, and septic shock. These symptoms may lead to the development of a lethal hyperglycemic crisis episode (HCE), such as diabetic ketoacidosis. Dong et al. investigated the risk factors of mortality in patients with DFUs-HCE. They developed a machine learning model, named XGBoost, to investigate and evaluate the risk factors of all-cause mortality in patients with DFU. The XGBoost model predicted dementia, osmolality, stock, serum albumin, chronic kidney disease, HCO^3-^, hemoglobin, and Hb1Ac to be the main risk factors of mortality in patients who developed DFU-HCE.

Diabetic retinopathy (DR) is another complication associated with T2D, which is dramatically heightened with longer disease duration and affects elderly patients. Calcium hydroxybenzene sulfonate is widely used for the treatment of DR, which acts by preserving retinal albumin, reducing capillary permeability, and inhibiting oxidative stress and aldose reductase. In addition, retinal laser photocoagulation, vitreous cavity injection of anti-vascular endothelial growth factor (VEGF), and steroid hormone drugs are also used for treating DR. In a systematic review, Li et al. compared the safety and efficacity of the conventional treatment modalities of DR with that of traditional Chinese herbal compounds (CHCs). The meta-analysis revealed that a combination of conventional medicine and CHCs significantly improved clinical parameters, such as efficiency, visual acuity, and fundus signs.

T1D, on the other hand, is a chronic autoimmune disorder that leads to the obliteration of pancreatic β-cells and is characterized by insulin deficiency and hyperglycemia. It is estimated that around 50% of the β-cells remain viable and functional at the onset of T1D. Initial studies by Nwosu revealed that vitamin D supplements are sufficient to protect the survival of β-cells, owing to its anti-inflammatory and immunomodulatory properties. Indeed, the study showed a significant reduction in the serum concentration of tumor necrosis factor alpha (TNF-α), a well-known pro-inflammatory marker. In the current issue, the author designed a clinical treatment protocol using a randomized controlled cohort model. At the onset of T1D, patients with low serum 25-hydroxyvitamin D_3_ (25(OH)D_3_; < 30 ng/mL) are recommended to initiate vitamin D supplementation, either ergocalciferol or cholecalciferol, to sustain serum 25(OH)D_3_ levels between 30 to 60 ng/mL. Whereas patients with high serum 25(OH)D_3_ (≥ 30 ng/mL) are encouraged to monitor their 25(OH)D_3_ serum levels periodically and sustain its concentration above the minimal limit.

Altogether, public awareness, further experimental studies, and drug discovery are key elements to control the development and progression of DM and its associated complications. Global campaigns aimed at raising awareness on DM should be frequently organized to disseminate information among the public about prediabetes, prevention of T2D, management of DM, disease symptoms, risk factors, and lifestyle modifications. Moreover, researchers and clinicians should be encouraged to conduct population-based studies using standard operating procedures.

## Author contributions

AA-M wrote the Editorial. HH revised the Editorial. All authors contributed to the article and approved the submitted version.

